# In normal rat, intraventricularly administered insulin-like growth factor-1 is rapidly cleared from CSF with limited distribution into brain

**DOI:** 10.1186/1743-8454-2-5

**Published:** 2005-07-26

**Authors:** Tavarekere N Nagaraja, Padma Patel, Martin Gorski, Peter D Gorevic, Clifford S Patlak, Joseph D Fenstermacher

**Affiliations:** 1 Department of Anesthesiology, Henry Ford Health System, Detroit, MI 48202, USA; 2 Department of Medicine, Mt. Sinai School of Medicine, New York, NY 10029, USA; 3 Department of Neurology, University of Pennsylvania, Philadelphia, PA 19104, USA

## Abstract

**Background:**

Putatively active drugs are often intraventricularly administered to gain direct access to brain and circumvent the blood-brain barrier. A few studies on the normal central nervous system (CNS) have shown, however, that the distribution of materials after intraventricular injections is much more limited than presumed and their exit from cerebrospinal fluid (CSF) is more rapid than generally believed. In this study, we report the intracranial distribution and the clearance from CSF and adjacent CNS tissue of radiolabeled insulin-like growth factor-1 after injection into one lateral ventricle of the normal rat brain.

**Methods:**

Under barbiturate anesthesia, ^125^I-labeled insulin-like growth factor-1 (IGF-1) was injected into one lateral ventricle of normal Sprague-Dawley rats. The subsequent distribution of IGF-1 through the cerebrospinal fluid (CSF) system and into brain, cerebral blood vessels, and systemic blood was measured over time by gamma counting and quantitative autoradiography (QAR).

**Results:**

Within 5 min of infusion, IGF-1 had spread from the infused lateral ventricle into and through the third and fourth ventricles. At this time, 25% of the infused IGF-1 had disappeared from the CSF-brain-meningeal system; the half time of this loss was 12 min. The plasma concentration of cleared IGF-1 was, however, very low from 2 to 9 min and only began to rise markedly after 20 min. This delay between loss and gain plus the lack of radiotracer in the cortical subarachnoid space suggested that much of the IGF-1 was cleared into blood via the cranial and/or spinal nerve roots and their associated lymphatic systems rather than periventricular tissue and arachnoid villi. Less than 10% of the injected radioactivity remained in the CSF-brain system after 180 min. The CSF and arteries and arterioles within the subarachnoid cisterns were labeled with IGF-1 within 10 min. Between 60 and 180 min, most of the radioactivity within the cranium was retained within and around these blood vessels and by periaqueductal gray matter. Tissue profiles at two sites next to ventricular CSF showed that IGF-1 penetrated less than 1.25 mm into brain tissue and appreciable ^125^I-activity remained at the tissue-ventricular CSF interface after 180 min.

**Conclusion:**

Our findings suggest that entry of IGF-1 into normal brain parenchyma after lateral ventricle administration is limited by rapid clearance from CSF and brain and slow movement, apparently by diffusion, into the periventricular tissue. Various growth factors and other neuroactive agents have been reported to be neuroprotective within the injured brain after intraventricular administration. It is postulated that the delivery of such factors to neurons and glia in the injured brain may be facilitated by abnormal CSF flow. These several observations suggest that the flow of CSF and entrained solutes may differ considerably between normal and abnormal brain and even among various neuropathologies.

## Background

Insulin-like growth factor-1 (IGF-1) is present in brain and cerebrospinal fluid (CSF) [1,2]. Its expression and the distribution of its receptors have been shown to change dynamically during development and differentiation [3], implying that IGF-1 is involved in these processes within the central nervous system (CNS). Hinting at some neuropathological role, CSF levels of IGF-1 have been shown to rise in several diseases, most notably with pituitary tumors [4,5]. Recently, IGF-1 has been used in some studies for its putative neuroprotective effects and has been suggested to be a potential therapeutic agent in many disorders of the nervous system including amyotrophic lateral sclerosis, Alzheimer's disease, and cerebral ischemia [6].

The routes of IGF-1 administration have varied among experimental studies and conditions, but intraventricular injections have often been employed, especially for the treatment of ischemic injury [7-12]. The intraventricular approach bypasses the blood-brain barrier (BBB) and implicitly assumes direct access of the injected material to most, if not all, brain tissue. Calling this assumption into question, however, are reports that show rapid, nearly complete clearance of intraventricularly injected radiolabeled sucrose, polyethylene glycol (PEG4000; MW = 4000 Da) and 40-amino acid amyloid peptide (Aβ 1–40) from CSF and brain into blood [13,14]. These studies also indicated that the small amount of radiolabeled material remaining in brain after 1–3 hr (<10% of injected) was mostly in the walls and/or perivascular spaces of the pial arteries and arterioles within the subarachnoid cisterns and in the tissue around the aqueduct of Sylvius [13,14]. The efficacy of delivery into brain via the CSF, has also been challenged by the finding that diffusion of higher molecular weight compounds from ventricular fluid into brain is restricted by the ependyma [15]. Smaller compounds (MW<5000 Da), however, readily permeate the ependyma, but their subsequent penetration into the parenchyma is limited by factors such as their restricted rate of diffusion through the tortuous interstitium, transcapillary loss, and cellular uptake and binding [16-18].

The neurobiological effects of intraventricularly injected substances are widely accepted, but the preceding observations suggest that the pathways, rates of distribution and sites of action within the neuraxis of such agents may be more complex, perhaps more limited, than assumed. Understanding the nature of such distribution may help explain the normal pathways of CSF flow and its entrained substances and the function and malfunction of this specialized brain fluid system, the so-called "third circulation."

In the present study, the distribution of ^125^I labeled IGF-1 (MW = 7430 Da) among CSF, brain, and blood between 2 min and 3 hr after a bolus intraventricular injection was investigated in rats. The hypothesis to be tested was that most of the intraventricularly injected peptide, IGF-1, would be quickly cleared from the CSF and brain into blood and that the remaining amount of IGF-1 would not be widely or uniformly distributed within brain.

## Methods

For these studies, ^125^I-IGF-1 (specific activity, 2 mCi/mmol; MW = 7430 Da) and ^125^I microscales were purchased from Amersham Life Sciences, USA. Male Sprague-Dawley rats weighing 300–350 g were obtained from Charles River (Cambridge, MA). All surgical procedures and experiments were performed according to National Institutes of Health guidelines under an approved protocol from the Institutional Animal Care and Use Committee of the Henry Ford Health System.

### Intraventricular injections

Intraventricular injections were made according to the methods previously described [13,14]. Briefly, one femoral vein and artery were cannulated under halothane anesthesia for subsequent injection of anesthetic agent (40 mg/kg; Nembutal sodium, Abbott Laboratories, Chicago, IL) and for sampling blood, respectively. After firmly positioning the head in a stereotaxic frame, 200–500 nCi of ^125^I-IGF-1 in either 0.5 or 1.0 μL saline was infused at the rate of 1.0 μL/min into the anterior horn of one lateral ventricle via a syringe pump (Sage Instruments: Model 351, Cambridge, MA). Stereotaxic coordinates for the infusion were: antero-posterior = 0.0 relative to bregma; lateral = 1.5 mm to the midline; and depth = 4.5 mm down from the surface of the skull. After infusion, the cannula was left in place for the duration of the experiment (n = 4–5 for each duration). Arterial blood samples were collected at pre-designated intervals. Animals were decapitated at times ranging from 2–180 min after injection, and the whole head was instantly frozen in 2-methylbutane cooled to minus 45°C.

Despite careful usage of the above stereotaxic coordinates, the infusate was sometimes delivered mainly into the brain parenchyma. Such infusions became obvious upon viewing the autoradiograms (ARG's). Any parenchymal injections (i.e., those missing the ventricle) were due to manual errors and were unintentional (n = 2–3). Data from such experiments were analyzed separately from those with intraventricular injections and some of these findings will be reported.

### Assessing organ and fluid radioactivity

The brains with accompanying CSF and blood plus surrounding meninges were dissected from the frozen head without thawing. Working within a chest freezer set at -20°C, the upper part of the skull was carefully removed with a rongeur. This frozen specimen contained virtually all of the intracranial contents and radioactivity. Immediately after removal, the frozen brain-meningeal-CSF specimens were placed in bottles chilled to -80°C and counted in a gamma counter (Wallac 1480, Turku, Finland). These data were used to calculate the clearance of ^125^I-radioactivity from the CSF-brain-meningeal system. Plasma was obtained from the blood samples, and radioactivity per unit volume of plasma determined. These samples were employed to track the appearance of radioactivity in blood following administration. Samples of urine, kidney, liver, and muscle were also assayed for radioactivity by gamma counting. In all instances, the counts were corrected for decay of ^125^I-activity. In a separate group of anesthetized rats (n = 4), subarachnoidal CSF was collected from the cisterna magna at several times after ^125^I-IGF-1 infusion. Acid precipitability of radioactivity was assessed on these specimens as well as on samples of plasma and urine from all the animals. All data obtained by gamma counting was calculated in units of dpm. Data obtained by quantification if autoradiographic images were calculated in nCi/g.

The procedure for obtaining the venous input rate, referred to hereafter as the emergence function, was similar to one used by Patlak and Pettigrew [19] to establish intravenous infusion schedules that yield specific arterial time-course [20,21]. First, a very rapid intravenous bolus injection was made and a carefully chosen series of well-timed blood samples were obtained: every 5–10 seconds for the first minute, every minute over the next 4 min, and then at longer intervals over the next hour or more. This curve depended on the distribution and clearance of the material throughout the body as well as the intravenous input function. A transfer function or transform that links the bolus intravenous injection to the experimentally determined arterial time-course was obtained. Then the reverse was done, namely, the transfer function was applied via the convolution integral to the arterial time-course measured after intraventricular infusion, thus generating the emergence function. In this operation, the transfer function took into account the whole body distribution and fate of the cleared material once it has entered the veins draining the site of injection including plasma protein binding and urinary excretion.

As just indicated, to obtain the emergence function, it was first necessary to determine the arterial time-course of radioactivity after a certain input into a systemic vein. To accomplish this, two rats were given an intravenous bolus of ^125^I-IGF-1 (~1.2 μCi). Eighteen blood samples of 20–25 μl each were drawn at a preset times between 5 sec and 60 min after the bolus. Plasma was obtained from the blood samples, and radioactivity per unit volume of plasma was determined.

### Autoradiography

After measuring total radioactivity as indicated above, the frozen brain-CSF-meningeal specimens were covered in cold embedding matrix (M-1; Lipshaw, Pittsburgh, PA) and stored at -80°C in sealed plastic bags until the time of sectioning. Using a cryostat set at -19°C and starting at the caudal end, the embedded brains were serially cut at 400 μm intervals into sets of five 20 μm thick sections. The first and fifth sections were picked up on a slide for subsequent Nissl staining; these histologies were used to identify the areas of interest. The second, third and fourth sections in the series were collected on chilled, numbered coverslips for autoradiography. The next fifteen sections were discarded, and the cycle was repeated up to the beginning of the olfactory tubercles. The coverslips were placed on a slide warmer at 60°C, which instantly dried and affixed the frozen sections. The coverslips plus a set of ^125^I-microscales (standards calibrated in nCi/g) were placed into an x-ray cassette along with a sheet of Kodak Biomax MR-1 film. After sufficient time of exposure (20 – 25 days), the films were developed.

### Image analysis

The autoradiographic images were digitized and visualized with an imaging system (Model AIS, Imaging Research Inc., St. Catharines, Canada). The matching Nissl stained sections were simultaneously displayed via a microfiche reader (Eye Communication Systems, Inc., Hartland, WI). Areas with appreciable amounts of radioactivity were located. These brain regions were demarcated on the histologies, and their exact location identified with a rat brain atlas [22].

Using the known values of the ^125^I-standards, a standard curve of optical density versus radioactivity in nCi/g was constructed for each autoradiograph. The optical densities of the tissue sections on the autoradiographs were converted into radioactivity units (nCi/g) using the standard curve and the radioactivity for the regions of interest was calculated. As indicated above, the quantity of injected radioactivity was varied among the experiments, with the greater amount infused for the longer times. This procedure led to adequate levels of radioactivity for accurate assaying in all experiments. For data analysis and presentation, all the values were normalized to 50 nCi per initial infusion.

To determine the distribution from lateral ventricle and aqueduct into brain, profiles of tissue radioactivity were constructed. A rectangle was drawn on the digitized image perpendicular to the ependymal border and extending 2 mm into the adjacent tissue. Radioactivity along this rectangle was measured at every 0.25 mm and a plot of radioactivity versus the distance was created. The areas under the curve for such plots were calculated using the trapezoid rule.

All data are reported as the mean ± SD.

## Results

### Clearance of ^125^I-IGF-1 from CSF and brain

**Figure 1:**
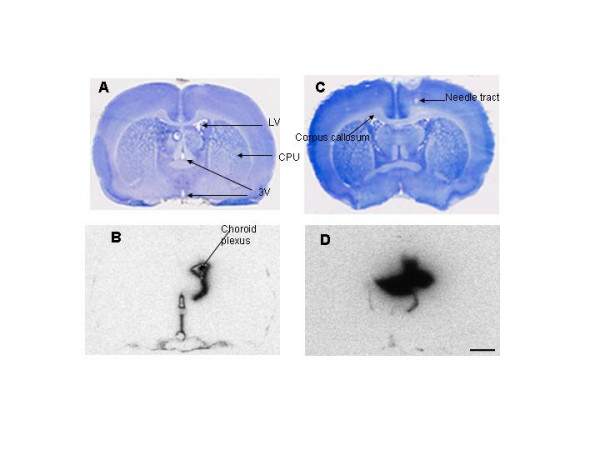
**Nissl-stained histologies (A and C) and adjacent autoradiograms (ARG's; B and D) showing the lateral (LV) and the third (3 V) ventricles 30 min after injection into one lateral ventricle (A and B, respectively) or into the parenchyma (C and D, respectively). 1A. **This histological section is at bregma -0.2 mm and cuts through the cerebral cortex (not labeled), LV, caudate-putamen (CPU), 3 V, anterior commissure (not labeled), and the optic chiasm (missing on the histology, but evident on the ARG, 1B). **1B. **The lateral ventricle choroid plexus and the walls of the LV and 3 V on the ipsilateral side are darkly labeled on this ARG, but little radioactivity is evident in the ventricles. The flattened loop of moderate darkness at the very bottom of the ARG demarcates the subarachnoid space around the optic chiasm. The dark half-ring or crescent above the optic chiasm partially surrounds the medial nucleus of the preoptic area, a peculiar pre-hypothalamic structure; this crescent appears to be an extension of the ventral part of the 3 V but may be an artifact. **1C. **This section is at bregma -0.3 mm and passes through the same structures as 1A above. Notable on this histology are the needle tract and corpus callosum, which on the ipsilateral side below the tract is expanded, pale, and edematous. **1D. **Most of the radioactivity in this ARG is contained in the tissue near the main site of the intraparenchymal infusion, but some is in the choroid plexus and CSF (both shown by the tail below the main patch of blackness on the ARG). Much of the tissue radioactivity is in the corpus callosum and some of it extends via this white matter tract across the midline. Scale bar = 1.5 mm.

**Figure 2:**
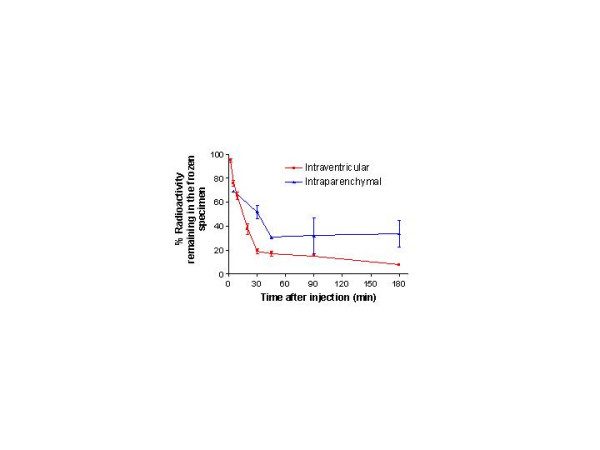
**The percentage of radioactivity remaining in the entire system of frozen brain, blood, CSF, and meninges over time following either intraventricular or intraparenchymal infusion of ^125^I-IGF-1. **There were 4–5 experiments per mean and time for the intraventricular infusions but only 2–3 for the intraparenchymal ones, which were accidental and not intended. Data are shown as mean ± SD. The initial phase dominated the first 30 min of clearance and accounted for most of the loss from the system for both sets of infusion data. From 45 min onward, virtually no radioactivity was lost from the system with the intraparenchymal infusion, whereas ^125^I-activity declined very slowly with intraventricular infusion (40% decline from 45 to 180 min).

Following accurate intraventricular administration (Fig. 1A, B), total intracranial radioactivity (brain-CSF-meningeal specimens) at 2 min after administration was approximately 93% of the amount infused, indicating complete or near complete delivery (Fig. 2). About 25% of the injected label was cleared from the system during the first 5 min and nearly 80% by 30 min. During the next 150 min, an additional 12% was cleared. When the infusion into one lateral ventricle was on target, the disappearance of ^125^I-radioactivity from the intracranial compartment was seemingly biphasic with half times and clearance fractions of 10 and 180 min and 79% and 21%, respectively. Almost all the radioactivity in the CSF drawn from the live, anesthetized rats was TCA-precipitable and presumed to be linked to IGF-1.

In 12 of 75 infusions, much of the radioactivity was accidentally deposited directly into the parenchyma (Fig. 1C, D). In these cases, the subsequent clearance of ^125^I-activity from the brain-CSF-meningeal specimen was different than that following direct intraventricular administration (Fig. 2). The data from these 12 rats suggest a rapid and sizable decrease (30% loss) of intracranial radioactivity over the first 5 min (the first time of sampling for this group of rats; n = 1) and a slower but larger decrease (40% loss) over the next 40 min. It is likely that most of this clearance was of IGF-1 delivered into the CSF. There was essentially no further loss of radioactivity from the brain-CSF-meningeal system over the remaining 135 min (Fig. 2). During this phase, most of the radioactivity was located in the tissue around the injection site dorsal to the lateral ventricle. Infusion into the parenchyma caused some swelling of the corpus callosum, the white matter structure that received much of the infusate (Fig. 1C). These observations are indicative of the distribution and tissue injury obtained when agents and tracers are deliberately or accidentally administered into the parenchyma [23,24].

**Figure 3:**
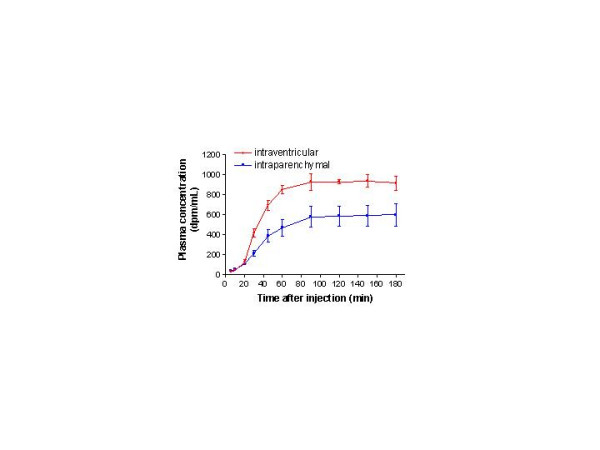
**Plasma radioactivity over time after infusions into the lateral ventricle and parenchyma. **Data are shown as mean ± SD. Plasma concentrations were fairly similar for both infusions up to 20 min, when the intraventricular infusion began to yield higher radioactivity; plasma concentrations for intraventricular infusions were about 50% higher than those for intraparenchymal infusion at 90–180 min. These data plus those in Fig. 2 clearly show the much greater clearance of intraventricularly infused ^125^I-IGF-1.

With both intraventricular and intraparenchymal infusions, low but significantly greater than background amounts of radioactivity were detected in the blood at 5 and 9 min (Fig. 3). Plasma ^125^I-activity rose sharply after 20 min in both cases and levelled off after 90 min, albeit at a much lower concentration for the intraparenchymal than intraventricular infusions (600 and 900 dpm/ml, respectively). Between 90 and180 min, the concentration in blood was essentially constant.

**Figure 4:**
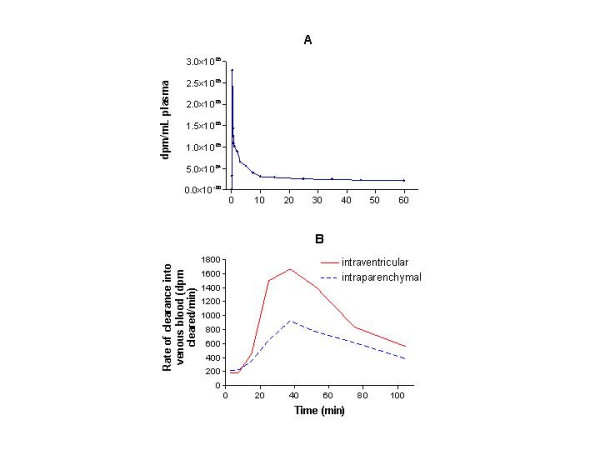
**Calculation and graphing of the emergence function, i.e., the rate of appearance of ^125^I-activity in venous blood. 4A. **The time-course of radioactivity concentration in arterial plasma after an intravenous bolus injection of ^125^I-IGF-1. **4B. **The emergence function calculated from the arterial plasma concentration-time curve following intravenous injection plus the arterial plasma curves following intraventricular and intraparenchymal infusions. The emergence function is the rate of appearance of the radioactivity in the venous system (dpm/min) and was calculated for 2.5, 7.5, 15, 25, 37.5, 52.5, and 75 min; the breakpoints on the graphs demarcate these times after infusion. The curves were not smoothed although the rates continually change throughout the period of interest.

For the arterial time-course following an *intravenous *bolus of ^125^I-IGF-1 (required to find the emergence function along with the preceding data), plasma radioactivity had begun to rise by 15 sec, reached a peak around 25 sec, dropped precipitously over the next several min, and slowly declined from 10–60 min (Fig. 4A). The rate of ^125^I-appearance in venous blood (emergence function, dpm per min) was low at 5 min (the earliest time with significant plasma radioactivity) but it was higher for intraparenchymal (221 dpm/min) than intraventricular (185 dpm/min) injections (Fig. 4B; rate given on the ordinate). After 7.5 min, the rate increased markedly, reaching a peak at approximately 40 min of 1665 dpm/min with intraventricular injection and 924 dpm/min with intraparenchymal administration. The emergence rates fell continuously thereafter, but good estimates beyond 60 min were not possible because of the low radioactivity and the flatness of the post-bolus blood curve from 45 min onward (Fig. 4A).

The lag between the rapid disappearance from the brain-CSF-meningeal specimen from 2 min to 30 min (Fig. 2) and the marked rise in blood concentration after 20 min (Fig. 3) is a bit surprising. This discrepancy suggests that most of the ^125^I-IGF-1 clearance from the intracranial compartment did not directly and immediately pass into blood across the capillaries of the structures around and within the ventricles, such as the choroid plexuses, subependymal zone, and circumventricular organs (e.g., the subfornical organ and median eminence).

**Figure 5:**
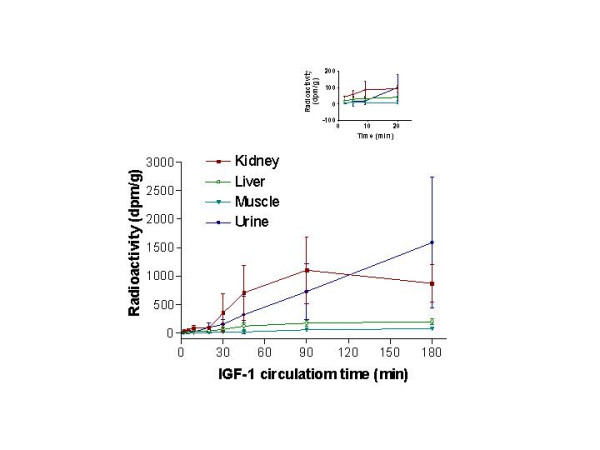
**IGF-1 radioactivity in kidney, liver, skeletal muscle and urine after its intraventricular injection. **Kidneys were always seen to preferentially sequester IGF-1 from blood in the first 90 min after injection. Appreciable amounts of radioactivity began to appear in the urine after an initial lag period of ~5 min. After 20 min, radioactivity in urine rose almost linearly with time whereas that in the kidneys began dropping after 90 min. Liver and skeletal muscle had comparatively very little radioactivity throughout the period of study. The inset shows the tissue radioactivity for the first 20 min that may not be evident on the main graph. All data are mean ± SD at the various times.

Among the three tissues sampled and urine, ^125^I-activity was at or near background at 2 and 5 min in all four (Fig. 5) and remained very low up to 20 min for liver, muscle, and urine. The radioactivity was somewhat elevated in the kidney at 9 and 20 min and thereafter arose slightly in the liver and much more in the kidney, and urine. Uptake of ^125^I by muscle, the largest organ in the body, was slow and slight. None of the ^125^I-activity in urine was TCA-precipitable, but 30–50% of that in plasma was. These findings demonstrate that little of the ^125^I-activity cleared from the brain-CSF-meningeal samples over the first 10 min passed immediately into blood or into non brain tissues and urine. Therefore the low plasma levels of radioactivity from 2–9 min (Fig. 3) were not the result of rapid systemic tissue uptake and urinary excretion.

### Distribution of IGF-1 radioactivity within the CSF system

**Figure 6:**
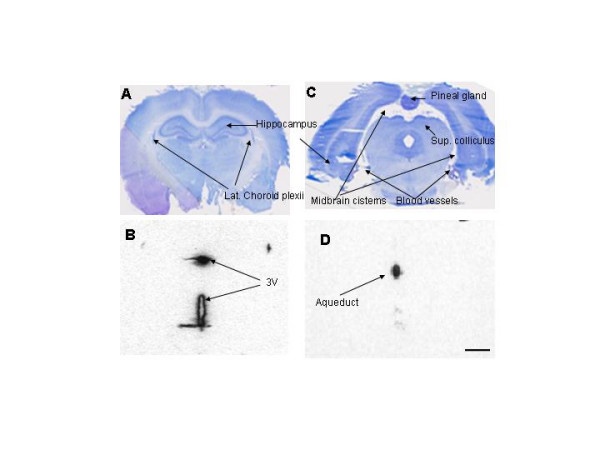
**Nissl-stained histologies (A and C) and adjacent autoradiograms (ARG's; B and D) showing the third ventricle (A and B, respectively; bregma -3.3 mm) and aqueduct of Sylvius (C and D, respectively; bregma -7.6 mm) 2 min after infusion of ^125^I-IGF-1 into one lateral ventricle (LV). 6A. **This histological section cuts through the parietal cortex, hippocampus, and thalamus (the large, light gray mass below the hippocampus) as well as the third ventricle (3 V), which is torn at its ventral border. The latter is formed by the very thin median eminence, which is one of the circumventricular organs and often tears during sectioning. **6B. **The ARG indicates considerable delivery of radioactivity to the dorsal (very dark and very full) and ventral parts of the 3 V within two min of ending the infusion. The lateral streak of black to the left of the dorsal 3 V is actually part of the latter that extends over the stria medullaris thalami, the white matter that forms the dorso-medial border of the thalamus. Some spreading of ^125^I-activity into the tissue adjacent to the ventral part of the 3 V is evident on the ARG at this time. The most ventral portion of this part of the 3 V flairs over the median eminence; the CSF in these little pockets explains, in part, the dark streaks radiating from the bottom of the ventral part of the 3 V and over the median eminence. The streak on the left seems, however, strangely long and has seldom been seen by us before. It may be real; it may be an artifact. **6C. **This histological section passes through the pineal gland, the superior colliculus (midbrain) and subiculum (cortical end of the hippocampus). The tiny dots surrounding the midbrain are clusters of small arteries and veins in the subarachnoid space and cisterns; at this level, the latter are the quadrigeminal (dorsal) and ambient (lateral) cisterns. **6D. **This ARG shows a very high level of radioactivity in the CSF within the aqueduct after only 2 min of circulation and indicates the speed at which CSF flows from the lateral ventricle through the 3 V system, which includes many recesses, and into the aqueduct. Scale bar = 1.5 mm.

**Figure 7:**
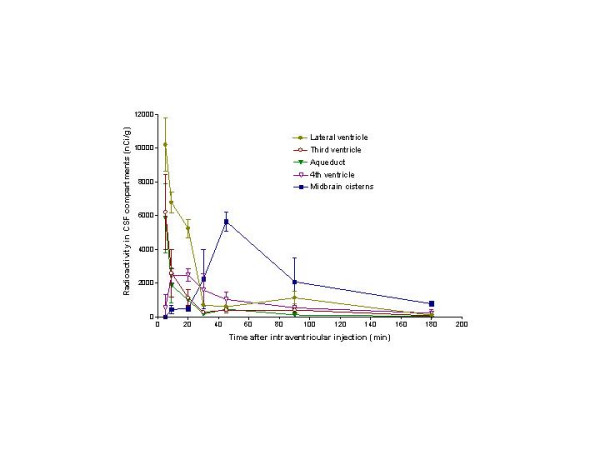
**Distribution of ^125^I-IGF-1 radioactivity in five CSF compartments as a function of time after ending the infusion. **The data are shown as mean ± SD at the various times. The first two sets of points are at 5 and 9 min. By 5 min, the peak concentrations have already passed through the lateral and third ventricles and the aqueduct; at 30 min, ^125^I-IGF-1 in these three compartments has fallen to <750 nCi/g. Radioactivity in the fourth ventricle reached a plateau over the 9–20 min period and dropped thereafter. In the midbrain cisterns, ^125^I-IGF-1 sharply rose from 20 to 45 min and then fell continuously thereafter. The peak radioactivity at 45 min is notable, exceeding that in the 4^th ^ventricle by 5-fold. These curves show that ^125^I-IGF-1 moved relatively briskly through the ventricular system.

Two min after infusion into one lateral ventricle, CSF-contained radioactivity was already present in the third ventricle (Fig. 6A) and aqueduct (Fig. 6B), but the optical densities at these two sites were too high for accurate quantitation. This was also true for the ipsilateral lateral ventricle. The concentrations of ^125^I-IGF-1 at 5 min were ~10,000 nCi/g in the CSF retained within the ipsilateral lateral ventricle and ~6000 nCi/g in that within the third ventricle and aqueduct (Fig. 7). Indicative of relatively rapid CSF flow, radioactivity fell sharply thereafter in these three CSF compartments, reaching low levels by 30 min, and becoming very low (<200 nCi/g) by 180 min. Between 2 and 5 min, most of the intracranial ^125^I-IGF-1 was present in the CSF within the lateral and third ventricles and the aqueduct. There was some early mixing of the injected radioactivity into the contralateral lateral ventricle (data not shown). The concentration of ^125^I IGF-1 within the fourth ventricle was appreciable at 5 min (Fig. 7), reached a maximum (>2000 nCi/g) at 10–20 min, and fell gradually thereafter.

**Figure 8:**
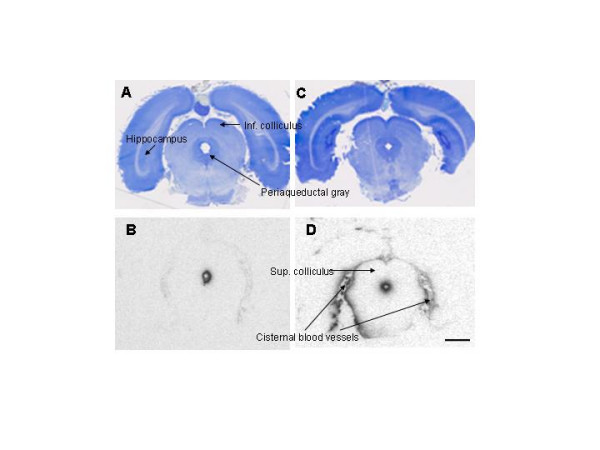
**Nissl-stained histologies (A and C) and adjacent autoradiograms (ARG's) at 20 min after ^125^I-IGF-1 infusion (A and B, respectively; bregma -7.8 mm) and 90 min (C and D, respectively; bregma -6.8 mm). 8A. **This section passes through the pineal gland, inferior colliculus (midbrain), aqueduct, and hippocampus and is just slightly caudal to Fig. 5C. Patches of small arteries and veins are scattered throughout the midbrain cisterns. **8B. **The adjacent ARG shows some penetration of radioactivity into periaqueductal gray matter at 20 min and a minute amount within the midbrain cisterns. **8C. **About 1.0 mm rostral to 7A, this section cuts through the superior colliculus, aqueduct, midbrain cisterns, dentate gyrus of the hippocampus, and the pontine nuclei (the large mass hanging below the midbrain). **8D. **The matching ARG, obtained at 90 min, indicates considerable retention of ^125^I-IGF-1 by the tissue immediately around the aqueduct and on both sides of the midbrain cisterns. The arteries and veins in the subarachnoid space also retain much radioactivity at this time. Scale bar = 1.5 mm.

Radioactivity in the quadrigeminal, ambient, and interpeduncular cisterns, parts of the midbrain subarachnoidal system, was essentially zero for the first five minutes but was detectable, albeit low, from 10–20 min (Figs. 7 and 8B); it rose rapidly from 20 to 45 min (~5700 nCi/g) and then fell over the next 135 min (Fig. 7). Over the 20–180 min period, much of the intracranial radioactivity was localized around the arteries and arterioles within these and other cisterns (Fig. 8A and 8B). This radioactivity could be in either the perivascular sheath, which contains CSF, or the vessel wall or both.

**Figure 9:**
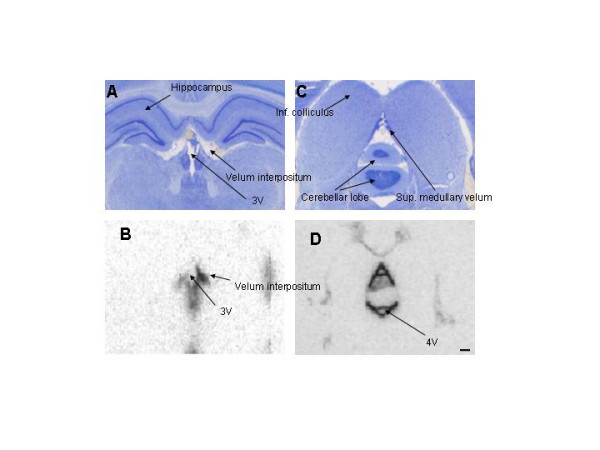
**Nissl-stained histologies (A and C) and adjacent autoradiograms (ARG's) at 30 min after ^125^I-IGF-1 infusion for the two subarachnoid velae, the velum interpositum (A and B, respectively; bregma -4.3 mm) and the superior (anterior) medullary velum (C and D, respectively; bregma -9.2 mm). 9A. **Starting at the subfornical organ and running caudad along the roof and upper sides of the third ventricle (3 V), the velum interpositum separates the 3 V from the subarachnoid space within it. The 3 V is clear and featureless, whereas the velum interpositum is filled with typical arachnoid tissue, e.g., trabeculae and blood vessels. The thalamus is the brain structure comprising the lower half of the figure. 9B. At 30 min, there was some radioactivity in the 3 V and the contralateral velum (left side) and much more in the ipsilateral velum. The dark spot at the bottom of the ARG came from ^125^I-activity within the mammillary recess of the 3 V. **9C. **The superior medullary velum (SMV) forms the posterior wall of the recess of the inferior colliculus (this recess is an outpouching ofthe aqueduct and not shown) and the anterior roof of the fourth ventricle, mostly in the vicinity of the cerebellum; it contains subarachnoid CSF and tissue. **9D. **The very dark "A-shaped" figure in the middle of this ARG indicates radioactivity that has collected over 30 min in the CSF and tissue of the SMV. The grayness within the legs of the upright "A" indicates some diffusion into the cerebellar lobe from the SMV. The legs and crossbar of the inverted "A" in the lower middle of the ARG represent radioactivity within the SMV on the ventral side of the cerebellar lobe (the SMV is not visible on the histology at this magnification, Fig. 9C; it can be seen to be subarachnoid tissue in Fig. 6 of Ghersi-Egea *et al*. [14]. The lighter spot within the lower part of the inverted "A" arises from CSF signal within the fourth ventricle. The faint figures in the rest of this ARG indicate radioactivity in several midbrain cisterns of the subarachnoid system at 30 min. Scale bar = 0.6 mm.

As we have reported for ^14^C-sucrose, ^14^C-PEG4000, and ^125^I-amyloid β peptide (Aβ1–40) [13,14], the radioactivity in the midbrain subarachnoid cisterns during the first 20 min appeared to come mostly from the third and fourth ventricles via the velum interpositum (Fig. 9A) and anterior medullary velum (Fig. 9B), respectively. These two velae are extensions of the subarachnoid space and form part of the membranous covering of these two ventricles. Once the CSF-entrained radioactivity moved from ventricle into these velae, it flowed from the velum interpositum and the superior medullary velum into the quadrigeminal, ambient and the interpeduncular cisterns. The sharp rise in midbrain cisternal ^125^I-IGF-1 from 20–45 min (Fig. 7) probably is mainly due to CSF flow via the "classic" route; that is, from the lateral recesses of the fourth ventricle through the foramina of Magendie and Luschka and into the cisterna magna and the subarachnoidal cisterns around the brain stem.

At longer times (e.g., 90 min; Fig. 8D), an appreciable uptake and retention of ^125^I-IGF-1 was evident for some of the tissues adjacent to the array of midbrain cisterns; among these tissues are the medial geniculate (a thalamic nucleus), the basal part of the cerebral peduncles, and the dentate gyrus of the hippocampus. Again as in our other studies, no radioactivity could be seen in the subarachnoid space over the cerebral cortices.

### Periventricular and periaqueductal tissue profiles

**Figure 10:**
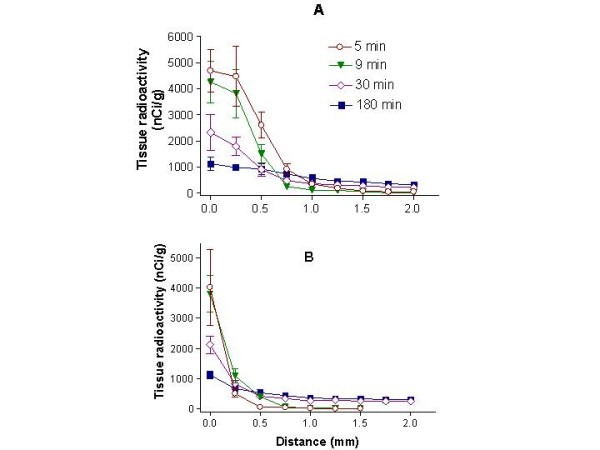
**Tissue radioactivities as a function of distance (profiles) away from the CSF-brain interface for the caudate-putamen (A) and periaqueductal gray matter (B) at four times. **The shapes of the curves and the concentration-distance integrals indicate two different tissue distribution dynamics. To illustrate this, we will consider only the 5 and 9 min profiles and avoid the matter of sizable tissue uptake from blood that affects the later times. For the caudate-putamen (10A), the 5 min points all lie above the 9 min ones out to 1.5 mm, where they both approach zero. The areas under these curves are 2757 (nCi/g) × mm at 5 min and 2002 (nCi/g) × mm at 9 min; clearly there was a loss of radioactivity, probably back into the CSF, over the 5–9 min period. In contrast, periaqueductal gray matter radioactivities (10B) were similar at the edge of the tissue (x = 0) at these two times but the 9 min points were higher than the 5 min ones from 0.25 to 0.75 mm, where both became essentially zero. The area under the 5 min curve was less than that under the 9 min one, 662 vs. 873 (nCi/g) × mm, respectively; periaqueductal gray matter, thus, continued to take up ^125^I-IGF-1 even as concentration in aqueductal CSF was falling (Fig. 7). One explanation for this is greater binding or trapping of IGF-1 in periaqueductal gray matter than in caudate-putamen. Data are shown as mean ± SD.

The tissue profiles of radioactivity indicated that the ependyma is not a significant barrier to the movement of IGF-1 from CSF into gray matter. This is evidenced by the sizable amounts of radiolabel that moved within the first 5 min as far as 500 μm into periventricular tissue, exemplified in this study by the caudate-putamen (Fig. 10A), and 250 μm into periaqueductal gray matter (Fig. 10B). These distances are reasonable for free diffusion through the tortuous extracellular space of the brain for such a duration and molecule. The differences in the amounts of radioactivity and its spread into the parenchyma between these two areas is caused by the 1–2 min delay in delivery to the aqueduct and the lower ^125^I-concentration within it (Fig. 7). The latter is the result of dilution within the third ventricle by inflowing unlabeled CSF from the contralateral lateral ventricle and by production of fresh CSF by the third ventricle choroid plexus.

As the CSF concentration falls within the ventricular system over time, the profiles flatten for both tissues sites. This decline is driven by a combination of backflux of unbound radiolabel from tissue to adjacent CSF and movement further into the tissue (see the legend to Fig. 10 for more on this). At all times and both tissue sites, ^125^I-activity decreased over distance into the brain and reached a plateau around 1.5–2.0 mm for caudate-putamen (Fig 10A) and 1.0–1.5 mm for periaqueductal gray matter (Fig. 10B). Indicating a net loss of IFG-1 over time, the mean area under the tissue radioactivity-distance curve over 2.0 mm for the caudate-putamen was highest at 5 min (2757 nCi mm g^-1^) and fell continuously thereafter reaching 1293 nCi mm g^-1 ^at 180 min. In contrast, the mean area under the curve for periaqueductal gray was lowest at 5 min (652 nCi mm g^-1^), rose to 955 nCi mm g^-1 ^by 30 min, and remained high at 180 min (922 nCi mm g^-1^). Regional differences in the uptake and retention of IGF-1 are obvious from these two tissue profiles.

## Discussion

### Experimental procedure

The intraventricular route of IGF-1 administration was selected because it has been used for many studies. Bolus injections into one lateral ventricle of IGF-1 have been reported, for instance, to improve neurological outcome for both permanent and transient cerebral ischemia in rats and other small laboratory animals [9-12]. A small bolus was chosen for injection in order to cause a minimal and transient disturbance of CSF pressure and flow.

Freezing the whole head immediately after decapitation has been shown to prevent collapse of the ventricles and preserve *pre-mortem *blood and CSF distribution within the cranial cavity [25]. A key part of this procedure is keeping the head and its contents frozen while removing the brain-CSF-meningeal specimen. The procedure is arduous, but these conditions minimize loss and *post-mortem *movement of radioactivity. In contrast to our procedure, others have removed the brain before freezing [15,26] or have perfusion-fixed the brain before sectioning and autoradiography [27].

The emergence function is based on the assumption of tracer kinetics; that is, the concentration of the radiotracer is presupposed to be very low with respect to the cold substrate, in this case, IGF-1. With this assumption, the distribution of ^125^I-IGF-1 is considered to be a linear function of its concentration in CSF, brain, and blood. There are IGF-1 binding proteins (IGFBP's) in the CNS and blood [6] and undoubtedly a state of dynamic equilibrium exists between IGF-1 and its binding proteins. Although ^125^I-IGF-1 concentrations were somewhat high in the ventricular and intravenous infusates, it would be diluted to tracer levels in plasma within seconds as it mixes in the circulating blood and binds to plasma proteins such as the IGFBP's. The tracer assumption for the intravenous infusion may be violated for the first 30 seconds or so (systemic circulation time in the normal rat is about 15 sec) but not thereafter. This dilution-mixing process is probably slower in the CSF, but the amount of ^125^I-IGF-1 intraventricularly infused was considerably lower, namely, around 25% of the intravenous dose, and that enhances the tracer assumption on the CNS side.

### Peculiarities in cerebrospinal fluid flow

Recent experiments indicate that about 25% of CSF and CSF-borne radiolabeled sucrose [14], PEG4000 [13], soluble amyloid beta peptide (sAβ1–40) [13], and IGF-1 (Fig. 9) flow into the subarachnoid extensions of the velum interpositum (from the dorsal part of third ventricle) and superior medullary velum (from the rostral part of the fourth ventricle). Within 4–5 min, intravelar CSF appears in the basal and midbrain cisterns and the arteries and arterioles contained within them [14]. The rest of the CSF flows from the sites of production within the ventricles to and through the lateral recesses of the fourth ventricle and the foramina of Luschka and Magendie into the cisterna magna. From there, this CSF passes into the spinal and cranial subarachnoid space. It was also noted in these three studies that very little of these radiolabel materials reached the cortical surface of the normal rat brain even after three hours of circulation.

Within the basal cisterns, all four of these radiolabeled materials accumulate in the pial arteries and arterioles. Anatomically, these blood vessels seem to be surrounded by a specialized pia-arachnoid membrane complex that functions to trap CSF-borne substrates. In addition, the highly vascular choroid plexuses were also observed to take up and retain some IGF-1 for the first 30 min (Fig. 1B and 5B). It should be noted that IGF-1 receptors are present in rat choroid plexus [28]. It may be that one or more of these vascular tissues are involved in the putative neuroactive effects of IGF-1 (Figs. 1 and 7).

Of relevance to vascular involvement, gene transfer to the adventitia and leptomeninges of arteries and arterioles along the ventral surface of the brain has been accomplished in mice via a recombinant adenovirus injected into either one lateral ventricle or the cisterna magna [29,30]. Such transfer was not, however, achieved on the dorsal surface of the brain, which may be due to the very low delivery of CSF-entrained substances over the cortex as observed in other studies [13,14] as well as the present one.

### Differences in the CSF-brain-blood distribution of peptides

Before proceeding further, an evaluation of the robustness of these techniques and data is in order. The gamma counting technique for the plasma and tissue radioactivity is very straightforward and highly accurate. The delay of several minutes before the elevation of both the plasma (Figs. 3) and systemic tissue (Figs. 3, 4, 5) ^125^I-activity is probably not an artefact. One possibility to consider is that not all of the IGF-1 reached the lateral ventricle CSF at injection and the initial rapid decline is caused by that shortcoming. At 2 min, however, the earliest time of sampling, around 93% of the radioactivity was still in the system, and most of the decrease occurred subsequently. Similar studies with ^14^C-sucrose [14] and ^14^C-PEG4000 [13] showed that virtually all the infused radioactivity was present within the cranium for the first 5 min. On the occasions when it was obvious from the autoradiograms that the IGF-1 was partially or completely infused into the brain parenchyma, those experiments were eliminated from the successful group. It, thus, seems that there is little or no problem with the accuracy of the intraventricular infusion method.

In the present study, IGF-1 exited from the brain-CSF-meningeal compartment at an unexpectedly fast rate. The clearance of ~50% of it by 15 min and ~80% by 30 min is far faster than that of sucrose [14] or PEG4000 [13] which had no clearance over the first 5 min and apparent half-lives ~60 min for both. Of the molecules studied to date, only ^125^I-labeled soluble amyloid β peptide (I-sAβ1–40) with a ~50% loss within 8 min [13], is cleared more rapidly than IGF-1. In another study, the half time of disappearance from brain of insulin has been calculated as 26 min in mice [26].

Rapid clearance of IGF-1 and I-sAβ1–40 from the brain-CSF-meningeal system would be expected to take place directly into the circulating cerebral blood and lead to systemic distribution. In the study with I-sAβ1–40 [13], this was found to be the case: when arterial blood concentration of I-sAβ1–40 was measured 3.5 min after intraventricular infusion, about 30% of the infused I-sAβ1–40 had already been cleared from the brain-CSF-meningeal system, and the blood level of ^125^I was high. With further clearance over time, the concentration of I-sAβ1–40 in blood continued to rise slowly, increasing by 25% between 3.5 and 30 min. Consistent with this, the tissue levels of ^125^I-activity were appreciable in liver at 3.5 and 10 min and even higher in the kidney at these two times. Although emergence function analysis was not done on these data, it was evident that the I-sAβ1–40 cleared from the CSF-brain-meningeal system passed immediately into the circulating blood and distributed throughout the body. Because of the speed of this process, the clearance of I-sAβ1–40 was assumed to be directly across the capillaries of the choroid plexuses, the subependymal zone, and other periventricular structures such as the circumventricular organs. This mechanism has been suggested for not only sAβ1–40 but also other peptides and immunoglobulins [13,31,32].

In contrast, this study found an unexpected delay between the loss of IGF-1 from the CSF-brain-meningeal system and its appearance in blood. Although 35% of the infused radioactivity was gone from the system after 9 min, the plasma concentration was very low (less than 8% of the maximum) at this time and at the preceding times of sampling (Fig. 3). Thereafter the clearance of IGF-1 from the CSF-brain-meningeal system continued, with only 20% remaining by 30 min. Plasma radioactivity increased between 9 and 20 min, more sharply between 20 and 30 min, and even more steeply between 30 and 45 min, finally reaching a plateau at 90 min (Fig. 3). Reflecting this delayed, slow increase in blood radioactivity, the emergence rate for IGF-1 (Fig. 4) began to increase between 9 and 12 min and then climbed markedly between 12 and 20 min.

A study on the clearance of a biologically inert and extracellular compound ^14^C-PEG4000 (MW = 4000 Da) following intraventricular infusion [13], showed that the compound was retained in higher concentration in the CSF system at early time points than for IGF-1, but a similar delay occurred before it appeared in blood. Since the PEG clearance pathway is probably non-specific and it is most likely lost by CSF bulk flow and absorption, it is possible that a sizable portion of the infused IGF-1 could also have been cleared via this route. However, the delay in appearance in blood in both studies indicates that the compounds lost from the brain-CSF-meningeal system at the early time points did not go directly into the circulating cerebral blood.

Hence the temporal mismatch of IGF-1 clearance from the CSF-brain-meningeal and its appearance in systemic blood remains enigmatic. A small part of the initial clearance of IGF-1 does seem to be directly into blood via the periventricular tissue and choroid plexuses as occurs with sAβ1–40. A much larger portion of it may flow rather rapidly out of the cranial space via the PEG-CSF pathway. The time-course and magnitude of this is suggested by the steep rise in the rate of appearance in systemic blood that starts after 9 min and peaks at 40 min (Fig. 4B). Much of this bulk CSF clearance may be by way of the cranial nerve sheaths and the cranial and cervical lymphatic systems as has been shown for albumin [33,34] or by passage from the cisterna magna into the spinal subarachnoid space, which is outside of our sampling field, and hence into the venous system. One or the other or both of these putative routes could be the physiological explanation of the delay in the IGF-1 appearance in blood.

### Tissue profiles

The tissue profiles showed some penetration of IGF-1 into the brain immediately adjacent to the CSF (Fig. 9A, caudate-putamen; Fig. 9B, periaqueductal gray matter) over the first 9 min when blood-contained radioactivity was low (Fig. 3). At these times, little or no radioactivity had, however, moved beyond 1.0 mm in the caudate-putamen and 0.5 mm in periaqueductal gray matter. At 30 and 180 min, the concentrations of radioactivity in the ependymal and subependymal tissue were much higher than in the adjacent CSF (Fig. 6), and the radioactivity at the peripheral zone was three-times higher than the deeper tissue plateau concentration. Collectively, these observations suggest that ^125^I-IGF or some labeled metabolite(s) is "trapped" in this tissue, perhaps to a receptor or within some periventricular intracellular compartment.

The implication of this is that for intraventricularly injected IGF-1 to be centrally effective it either interacts with receptors or compartments that are immediately around the ventricular system or works at deep active sites at the low levels indicated by the 30–180 min ^125^I-IGF-1 plateaux (Fig. 9). Recent work has shown the presence of neuronal progenitor cells in the subependymal layer (a.k.a., the subventricular zone), especially of the lateral ventricles and dentate gyrus of the hippocampus [35,36]. Such cells are well positioned with respect to circulating CSF and could be the target of CSF-borne growth factors.

### Intraventricular IGF-1 delivery following brain injury

Injury to the brain might result in changes to the flow of CSF and affect the distribution of intraventricularly administered peptides into brain. For example, 2 hr after hypoxic-ischemic injury, tritiated IGF-1 was intraventricularly infused for 30 min and immediately thereafter was found in the ipsilateral cerebral cortex [37]; this cortical radioactivity decreased over the next 6 hr and reached background (the contralateral level) after 12 hr. Microautoradiography indicated that ^3^H-IGF-1 was also present in the corpus callosum and its ventral extension, the external capsule, plus the perivascular (Virchow-Robin) spaces that surround the penetrating arteries and arterioles of the cortex 30 min after ending the infusion. This contrasts with our findings for normal brain of little or no IGF distribution to the cerebral cortex and underlying white matter. On the other hand, in our experimental "mistakes" in which much of the infusate was placed in the parenchyma causing trauma, the white matter was also heavily labeled with ^125^I-IGF-1 at all times of sampling (30 min data showed in Fig. 1D).

As to clearance into systemic blood, the serum concentration of ^3^H-IGF-1 in the studies of Guan *et al *[37] was high at 30 min after beginning the infusion in both hypoxia-ischemia injured rats and uninjured controls and rose 3-fold and 5-fold, respectively, over the next 3 hr. This resembles the plasma data given in Figure 3 for the intraparenchymal (accidental) and intraventricular infusion groups.

In a later study with the same hypoxia-ischemia model and intraventricular infusion protocol, Guan *et al *[38] investigated the cellular distribution of ^3^H-IGF-1 and its co-localization with immunoreactive IGFBP-2 at 30 min and 6 hr after administration. Thirty min after ending the infusion of ^3^H-IGF-1, it was mainly found in the pia mater, the perivascular spaces (extensions of the external subarachnoid space), and subcortical white matter structures such as the corpus callosum. The addition of cold IGF-1 to the infusate diminished the distribution of radiotracer to white matter tracts but not to the pia mater and perivascular spaces. Within subcortical white matter, tritiated IGF-1 co-localized with IGFBP-2 immunoreactivity and was seen at the microautoradiographic level to be associated with fiber tracts and some oligodendrocytes. In the cerebral cortex 30 min after completing the infusion, the ^3^H-signal was diffusely distributed but evident on the neurons of layers II-V, glia (astrocytes or microglia were not specified), and the extracellular matrix. On the basis of these data and the earlier study [37], the authors suggested for this model of hypoxia-ischemia that: 1) such patterns of IGF-1 distribution were not compatible with simple diffusion and were likely to be abnormal; 2) bulk flow of CSF through the ependyma and along white matter tracts and also through the subarachnoidal perivascular spaces carried IGF-1, in some cases in association with IGFBP-2, to the neurons and glia of the injured cerebral cortex; and 3) this abnormal flow of CSF promotes the delivery of intraventricularly administered therapeutic agents to damaged tissue sites.

Our findings are consistent with this postulate in several somewhat indirect ways. The observations on normal brain indicate little or no distribution of intraventricularly infused IGF-1 to subcortical white matter, cerebral cortex, and perivascular spaces over 3 hr (Figs. 1A, 1B, and 5); the patterns reported by Guan *et al *[37] are, thus, most probably abnormal and due to the pathological state. In support of this, in the experiments where the IGF-1 was infused into the parenchyma and injured the tissue, much of the radioactivity was found in the edematous corpus callosum (Fig. 1C and 1D). The data in Figure 9 show that IGF-1 does not move very far into normal gray matter and illustrate the limits of such movement, probably mostly diffusional, in control conditions. Seemingly, bulk flow of CSF plus any edema fluid formed within the parenchyma is needed to transport IGF-1 as widely as appears to be the case in the hypoxia-ischemia model [37]. Of some relevance to this, convection enhanced delivery of chemotherapeutic agents infused directly into the parenchyma at appreciable rates of volume flow are currently under investigation in several laboratories and neurosurgical settings [39,40].

### Metabolism of IGF-1 in brain

Finally, the retention of the radiolabel on the parent compound is always a problem in the interpretation of data from studies such as ours. Proteases in many organs of the body including brain have been shown to cleave IGF-1 into des(1–3)IGF-1 and the tripeptide, glycine-proline-glutamate (GPE) [41]. It is possible that one or more of these proteolytic products is no longer radiolabeled, is not rapidly cleared from the system, does not permeate the BBB, can diffuse great distances in brain tissue and reach the appropriate receptors, and is active. Fitting with this scheme, some reports have shown the beneficial effects of intraventricularly injected active IGF-1 fragment in experimental studies [42,43]. It was also felt that the tripeptide GPE was more effective than its prohormone IGF-1 [43]. If substantiated, then rapid removal of IGF-1 from the brain reported herein may be the reason for the lesser effectiveness of this growth factor.

## Conclusion

Further analyses of the normal flow of CSF and distribution of its entrained materials are needed for the comprehensive understanding of this system and its therapeutic potential. Such studies may shed light on more appropriate sites for intracerebral injections for accessing specific regions and pathways within the brain via this system.

## Competing interests

The author(s) declare that they have no competing interests.

## Authors' contributions

TNN performed all the intracerebral injections, blood sampling and QAR quantification studies and drafted the manuscript. PP carried out the QAR analyses. MG participated in the dissection of brains and plasma precipitation and measurements of radioactivity. PDG participated in the design of the study and funded the study. CSP performed the emergence function analysis. JDF conceived the study, participated in its design and coordination and helped draft the manuscript. All authors read and approved the final manuscript.
